# Tropical Trees Will Need to Acclimate to Rising Temperatures—But Can They?

**DOI:** 10.3390/plants12173142

**Published:** 2023-08-31

**Authors:** Kenneth J. Feeley, Manuel Bernal-Escobar, Riley Fortier, Alyssa T. Kullberg

**Affiliations:** Department of Biology, University of Miami, Coral Gables, FL 33146, USA; manuel.bernal.escobar@gmail.com (M.B.-E.); fortier.riley@gmail.com (R.F.); alyssa.kullberg@gmail.com (A.T.K.)

**Keywords:** adaptation, acclimation, species migrations, climate change, global warming, tropical forests, trees, biodiversity

## Abstract

For tropical forests to survive anthropogenic global warming, trees will need to avoid rising temperatures through range shifts and “species migrations” or tolerate the newly emerging conditions through adaptation and/or acclimation. In this literature review, we synthesize the available knowledge to show that although many tropical tree species are shifting their distributions to higher, cooler elevations, the rates of these migrations are too slow to offset ongoing changes in temperatures, especially in lowland tropical rainforests where thermal gradients are shallow or nonexistent. We also show that the rapidity and severity of global warming make it unlikely that tropical tree species can adapt (with some possible exceptions). We argue that the best hope for tropical tree species to avoid becoming “committed to extinction” is individual-level acclimation. Although several new methods are being used to test for acclimation, we unfortunately still do not know if tropical tree species can acclimate, how acclimation abilities vary between species, or what factors may prevent or facilitate acclimation. Until all of these questions are answered, our ability to predict the fate of tropical species and tropical forests—and the many services that they provide to humanity—remains critically impaired.

## 1. Introduction

Despite covering only a relatively small fraction of the Earth’s surface [1], rainforests harbor myriad of known and unknown species [2,3] and support human livelihoods directly through the production of food and natural resources [4] and indirectly through a diverse range of other important ecosystem services such as carbon sequestration and climate regulation [5,6,7]. Unfortunately, anthropogenic global warming threatens the persistence of many tropical tree species [8] and perhaps even the persistence of tropical rainforests as a whole [9,10].

Given the extreme taxonomic, functional, and ecological diversity of tropical trees, trying to understand their responses to global warming (which is itself a complex phenomenon involving not just temperature but also the indirect and interacting effects of many other abiotic and biotic factors) is a daunting task. This challenge can be simplified by keeping in mind that regardless of their differences, all species share a limited number of options for responding to their changing environments. Specifically, species can avoid unsuitable temperatures through species migrations, or they can tolerate changes in temperature through evolutionary adaptation or physiological acclimation (see Table 1 for a glossary of key terms used in this review). Any species that fails to avoid or tolerate rising temperatures will experience decreased performance and may become “committed to extinction” [11].

In this review, we synthesize the available knowledge in an attempt to answer the following three questions:

(1) Can tropical tree speciesremain at equilibrium with anthropogenic global warming and rising temperatures through species migrations?
(2) Can tropical tree species adapt fast enough to tolerate rapidly rising temperatures?
(3) Can individual tropical trees acclimate to rising temperatures?

Based on our review of the published literature, we argue that most tropical tree species will not be able to migrate or adapt fast enough to keep pace with anthropogenic global warming, and thus the fate of tropical forests will depend most heavily on the ability of trees to acclimate to hotter temperatures.

*Question 1: Can tropical tree species shift their geographic distributions to remain at equilibrium with anthropogenic global warming*?

It has been previously said of plants that “It is easier to move than to evolve” [12,13]. Indeed, a growing body of research provides strong evidence that modern climate change is forcing many tropical tree species to shift their geographic ranges, or “migrate”, out of the areas that are becoming “too hot” and into areas that were previously “too cold” (e.g., to higher elevations), with important effects on community composition and ecosystem dynamics [14,15,16,17,18,19].

Palaeoecological studies based on pollen records indicate that during past climate change episodes, tropical trees were able to migrate fast enough to maintain climate fidelity [20]. However, it appears that tropical tree species can no longer “keep up”. Indeed, a growing number of studies indicate that the current migration rates of tropical trees are markedly slower than what is required to remain at equilibrium with modern anthropogenic warming [17,21]. For example, Fadrique et al. (2018) used plot-based forest inventories to document widespread changes in the functional composition of Andean tree communities consistent with upward species migrations. Specifically, they found that in the Andean plots with repeat censuses, the relative abundances of lowland heat-tolerant (i.e., “thermophilic”) species had increased over the past two decades (2000–2015), whereas the abundance of highland species had decreased (a pattern referred to as “community thermophilization”). The rates of compositional change observed in the Andes corresponded to an average species thermal migration rate of just 0.003 or 0.007 °C per year (equivalent to approximately 0.5 or 1.3 m elevation per year) depending on the methods and plots used, which was markedly less than the concurrent regional warming rate of 0.06 °C per year. Similar rates of community thermophilization have also been recorded for montane tropical forest plots in Central America [15] and the Caribbean [22]. Tree species migrations and compositional changes may be even slower in lowland tropical areas [23] where latitudinal temperature gradients are shallow or nonexistent [24,25,26] and thus distances between climate analogs are greatest [27,28].

The slow rates of species migration in the tropics, even in montane systems with steep temperature gradients, may be due to the natural dispersal limitations of the tree species. For example, in their study of Andean trees, Duque et al. (2015) found that forests with higher abundances of vertebrate- or wind-dispersed tree species had higher rates of thermophilization than forests with higher abundances of tree species dispersed by insects, water, or gravity [16].

Species migrations may also be impeded by abrupt changes in climate or at ecotonal boundaries where factors other than temperatures limit species distributions [29,30]. In many parts of the high tropical Andes, the upper elevational limit of tree growth (i.e., the treeline) has remained remarkably stable over the past several decades despite rapidly rising temperatures [31]. In the case of tropical montane treelines, the stability may be due to the effects of UV radiation, cold nighttime temperatures, cold snaps, or species interactions preventing the expansion or shift of the forest species’ ranges into higher elevations [32,33]. As a visually dramatic ecotone, the treeline has been the focus of numerous studies; other ecotones, such as those occurring at cloud base or where soil conditions change [34,35,36], may be equally important in setting current and future species’ distributions [29,30] but have received considerably less attention.

Another natural factor that may slow or prevent tree migrations is priority effects [37]. For a species to migrate and expand its range into new areas, it will have to compete with the established incumbent species [38]. Incumbent species may be at an inherent advantage and thus may be able to persist and prevent incursions by new species. For example, although elevated temperatures in an area may slow the growth of an incumbent tree species and/or prevent it from reproducing, any surviving adults may still prevent more thermophilic tree species from immigrating and establishing by casting deep shade, producing allelopathic chemicals, or simply by occupying space. If this is the case, then disturbances (landslides, fires, severe storms, droughts, heatwaves, etc.) that hasten the mortality of the established trees and thereby reduce priority effects may accelerate species migrations and compositional changes [39]. This will be especially true if the mortality caused by the disturbance is non-random and disproportionately affects species that are already stressed by the changing climate. For example, in Jamaica, the rate of thermophilization in montane forest communities was greatly accelerated in the years after a strong hurricane that elevated the mortality of highland tree species and subsequently allowed for an increased recruitment of the more thermophilic lowland species [22]. Given the premium on high dispersal and establishment rates, species migrations are likely to favor pioneer or early successional species [40].

In addition to these natural limitations to species migrations, humans are creating many additional obstacles. In the Amazon, forest loss, degradation and fragmentation are reducing the amount of suitable habitat available for species to migrate to and are also increasing the distances that species need to migrate to remain at equilibrium with the climate [28,41]. Indeed, it is predicted that for large parts of the Amazon, deforestation will soon sever all connections between analog climates, making it extremely unlikely that any resident tree species will be able to escape rising temperatures through migrations [28]. These problems will be exacerbated by defaunation [42,43] and will likely cause the migration of vertebrate-dispersed tree species to decelerate [44]—even as the rate of global warming continues to increase [45].

For the few tropical tree species that can migrate fast enough to keep up with rising temperatures, habitat specialization on factors beyond temperature (e.g., soil conditions and nutrient availability, water availability, topography, etc.) may prevent them from finding suitable habitats in the future or cause them to migrate in different directions [28,46,47]. As species migrate at different rates and respond to different environmental factors, the composition of tropical forests will become mixed, creating novel communities [48] which will impose new biotic challenges for the species as they are introduced to new pathogens, herbivores, and competitors [49,50].

Can tropical tree species migrate and avoid global warming? Although many tropical tree species are shifting their distributions to higher elevations in response to modern climate change, these migrations are almost always too slow to offset modern warming and, in the tropics, appear to be restricted to primarily montane ecosystems. As such, we must look to the potential mechanisms for tropical tree species to persist by tolerating novel conditions.

*Question 2: Can tropical tree species adapt their climatic niches and tolerate anthropogenic global warming*?

Evolutionary adaptation (hereafter referred to simply as ‘adaptation’) requires heritable changes in a population’s or species’ genotype across multiple generations (Table 1). Many rainforest tree species are long-lived, with average lifespans of several hundred years [51,52,53], and some individuals reaching ages of more than 1000 years old [54]. Furthermore, some rainforest trees can take decades to reach reproductive maturity [55]. Because adaptation, even at its fastest, requires many generations, the extreme speed at which global warming is now occurring (>2 °C warming per century) relative to the long lifespans and generation times of most trees precludes adaptation as a response [56,57,58]. Previous studies have shown that rates of thermal niche adaptation by plants (including even non-trees with shorter lifespans and generation times) after the last glacial maximum were many orders of magnitude slower than required to keep up with warming [59] and that evolution of high temperature tolerances is particularly slow due to intrinsic physiological constraints [60,61]. Indeed, tropical tree species and genera typically exhibit strong thermal niche conservatism with closely related taxa tending to occur at similar elevations or temperatures (even when comparing across hemispheres), suggesting that there are evolutionary constraints preventing their adaptation to new elevational/thermal niches, and that speciation occurs most often due to genetic isolation and/or adaptation to non-climatic factors [62,63,64,65,66]. In other words, tropical tree species could not, or at least did not, respond to past climate change through adaptation. Given that the Earth is now hotter than it has been for >125,000 years [67] and that temperatures continue to rise at rates that are at least an order of magnitude faster than past post-glacial warming and are unprecedented for at least the past 50+ million years (and possibly 250+ million years) [68,69], we contend that adaptation and evolutionary rescue [70,71] are not viable responses for most tree species to modern anthropogenic climate change.

There are some possible exceptions. If, as has been previously posited, plants do not have segregated germlines (Table 1), then flowers on different branches of long-lived trees could be separated by many generations of cell divisions and therefore may accumulate different somatic mutations [72]. This could potentially increase the genetic variability of a single tree’s offspring and thereby allow for accelerated evolutionary adaptation. Contrary to this idea, newer studies have found that many plants do in fact have ‘functional germlines’ [73,74,75]. This means that the reproductive cells in a tree are separated by fewer cell divisions, which protects them from accumulating somatic mutations and decreases intra-individual genetic variability (i.e., the unit of reproduction in plants is in fact the individual and not the branch or individual flower). Additional research is clearly needed to understand germline segregation in plants and its effects on adaptability.

A second potential mechanism for accelerated adaptation is that even if the trees themselves are slow to evolve, their microbial symbionts may be able to evolve much faster and help their partner trees “adapt” to changing conditions [76,77,78]. Studies have found that the tolerances of some plants to environmental stressors such as heat, drought, and salt can be determined at least in part through their interactions with endophytic and/or mycorrhizal fungal symbionts [79,80,81,82,83] and potentially even through the interactions of the fungi with their viruses [84]. Because these fungal symbionts have much shorter generation times, they may be able to quickly adapt and consequently confer greater tolerances to their partner plants. Alternatively, the plants may shift their interactions to favor symbiotic species that incur increased tolerances [85]. Inoculation experiments have demonstrated the potential for altered mycorrhizal fungi communities to strongly enhance stress tolerances of herbaceous crop plants [86] and temperate trees [85], but at this point, it remains unclear what role fungal symbionts play in determining the environmental tolerances of tropical trees, much less the potential for symbionts to enable tropical tree species to more-quickly adapt to rising temperatures [87].

Can tropical tree species adapt to anthropogenic global warming? Global warming is almost certainly too fast for most plant species to tolerate through evolutionary adaptations, and evidence for the evolution of increased stress tolerance through plant microbial symbionts is still lacking for tropical trees. Therefore, the survival of most tropical trees will likely depend on the ability of individuals to tolerate climate change through plasticity and acclimation.

*Question 3: Can tropical trees acclimate to rising temperatures*?

Given their long lifespans, many of the tropical trees alive today have already experienced >1 °C warming and may live to see an additional 1–2 °C global warming [88]. The rapid pace of climate change relative to trees’ lifespans necessitates that individuals acclimate to changes in climate (i.e., change their behavior, physiology, metabolism, and/or structure through phenotypic plasticity; Table 1) to maintain consistent performance and fitness.

Previous studies have attempted to test for the acclimation of plants to climate change by examining how performance and/or functional traits vary over natural environmental gradients (e.g., across latitude or elevation), which serve as “space-for-time” proxies for future climate scenarios [89,90]. If there are differences between the traits of individual plants growing in hot places compared to the traits of conspecific plants growing in colder places, this could suggest that species are capable of acclimating in response to rising temperatures. For example, by examining the leaf traits of conspecific trees growing across a temperature and moisture gradient in southern Brazil, Souza et al. (2018) found that variation in morphological and functional traits (i.e., plasticity of traits) was associated with differences in temperature, aridity, and light availability [91]. Slot et al. (2021) measured the photosynthetic heat tolerances of leaves from seven tree species growing at different elevations and temperatures in Panama and found that heat tolerances and critical temperatures decreased with elevation and increased with temperature in just over half the species [92]. An important caveat to any studies using large-scale gradients is that they typically include many confounding variables such as spatially patterned changes in precipitation, seasonality, soil type, co-occurring species, etc. [46]. Perhaps even more importantly, any observed differences in traits across large-scale gradients may reflect genetic differentiation and local adaptation of allopatric populations, rather than just the acclimation of individuals [93,94]. Indeed, most studies on tropical gradients focus on interspecific variation and only a much smaller number even attempt to assess intraspecific variation.

By combining gradient-based studies with common gardens or transplant experiments, it may be possible to decouple the roles of adaptation vs. acclimation in determining intraspecific trait variation (e.g., [95,96,97]). Likewise, in situ experimental warming experiments can be used to test for acclimatory responses to rising temperatures [98]. For example, in the Rwanda TREE project, individuals from 20 different tree species were experimentally transplanted into three montane locations spanning elevations corresponding to a ~6 °C gradient in mean annual temperature (coupled with water and nutrient availability treatments) [99,100,101,102]. After three years of monitoring, results from these transplants show mixed evidence for acclimation to higher temperatures. Studies testing for the acclimation of leaf functional traits and photosynthetic parameters in 18 of the species found differences in the responses of species to warming as well as between successional groups. Specifically, leaf area decreased with warming in the early successional but not in the later successional species, whereas decreases in leaf mass per area and increases in leaf width-to-length ratio were common in both species groups [99]. Net photosynthesis strongly decreased with warming in both sets of species under dry conditions, but only decreased under wet conditions in later successional species [100]. In another Rwanda TREE study of three species with different water use strategies and leaf morphologies, all of the species exhibited acclimation of photosynthetic heat tolerances linked to the saturation level of thylakoid membrane lipids when grown at higher temperatures. However, this acclimation was insufficient to offset higher leaf and air temperatures, leading to narrower thermal safety margins [101]. Finally, a Rwanda TREE study using two of the same species as above did not find evidence of acclimation in the species’ thermal optima of photosynthesis or other photosynthetic parameters, but did find acclimatory decreases in photosynthetic capacity and leaf respiration in trees grown at the warmer sites [102].

In an analogous transplant study in the Colombian Andes (Montane-acclim), individuals from 15 tree species with different thermal affinities (i.e., 11 cold-affiliated and 4 warm-affiliated species) were planted in three common-garden sites spanning a 2000 m elevation gradient corresponding to a ~12 °C gradient in mean annual temperatures. Five months after planting, measures of leaf photosynthesis and respiration showed that all tree species continued to perform best at the temperatures closest to their natural distributions but also showed some evidence of acclimation in photosynthetic and respiratory parameters (with patterns suggesting greater plasticity of photosynthetic capacity in the cold-affiliated vs. warm-affiliated species) [103].

In situ warming experiments have likewise produced mixed evidence for the acclimation of tropical trees to rising temperatures. The TRACE project in Puerto Rico applied a 4 °C warming treatment to understory plants in a wet rainforest using infrared heaters. Studies of the two most common species (both small understory shrubs) have shown contrasting results. One species responded to the experimental warming treatment through an increase in the temperature at which photosynthesis is optimized but did not show any evidence of acclimation of respiration. The second species also did not show any evidence of acclimation of respiration and actually exhibited decreased photosynthetic yields, possibly due to lower stomatal conductances under elevated temperatures [104]. In Panama, a 4 °C soil warming experiment (SWELTR) was used to test the effects of rising temperatures on photosynthesis and growth of seedlings of six rainforest tree species. After 3 years, overall growth rates were significantly lower for seedlings in the experimentally warmed soils, but the declines were only significant for two species, *Inga laurina* and *Tachigali versicolor*. Both of these species are N-fixers, suggesting that warming could have a strong negative effect on N-fixation [105].

The TRACE, SWELTR, Rwanda TREE, and Montane-acclim projects are all ongoing (along with several other transplant studies and field experiments to test the effects of changing water availability and other conditions (e.g., [106,107]). Unfortunately, these sorts of experimental studies (1) are generally not logistically or financially feasible for most tropical forests, (2) are limited to small scales and thus cannot include many species or species interactions (for example, the TRACE warming experiment includes three warming plots and three control plots, each of which are just 12 m^2^; the SWELTR experiment includes 10 paired control and heated plots, each of which is ~20 m^2^), (3) can usually only test for relatively short-term responses in smaller growth forms or early life stages (as it is logistically infeasible to transplant adult trees, wait for planted seeds to grow into adult trees, or manipulate the climate around large trees; for example, in the TRACE warming experiment, the heaters are <3 m above the ground meaning that only smaller understory plants are included), and (4) apply instantaneous or unrealistically fast warming rates [108,109]. These problems are further compounded in laboratory or chamber-based warming experiments, which can provide precise data on physiological responses to temperature and climate (e.g., [110,111,112]) but only for small numbers of isolated and small-statured individuals growing in artificial ex situ conditions. Given the observed differences between species (e.g., between cold- and warm-affiliated species in the Montane-acclim project, between early and late successional species in the Rwanda TREE project, and between N-fixers and non N-fixers in the SWELTR experiment) and given the large temporal and spatial scale over which community dynamics occur, in situ and ex situ experiments are unlikely to ever capture the full range of possible species or community level responses.

A potential way to overcome some of the intrinsic limitations of these experimental studies and to test for long-term acclimation in individual adult canopy trees is through longitudinal studies [94]. In longitudinal studies, traits of interest are measured repeatedly through time on individual trees as the climate changes concurrently. However, due to the relative recency of functional trait studies, the individual-level data required for long-term longitudinal studies are essentially non-existent for tropical trees. Indeed, to explore how functional traits have changed in individual trees due to climate change over the past decades, we would need to go back in time to measure functional traits in trees that are alive today; fortunately, there are at least two approaches that may allow us to do just this—tree rings and herbarium samples.

Annual tree rings are undoubtably less distinct and less widespread in the tropics than in temperate and boreal zones, but an increasing number of studies have demonstrated that many tropical tree species (i.e., >230 species; refs. [88,113]) do in fact exhibit visible annual growth rings (including in the wettest perhumid tropical forests [114]). Even in the absence of visible annual rings, it is possible to date wood samples and create chronosequences based on high-resolution isotopic measurements and/or radiocarbon dating [115,116]. Dated wood samples allow for records of individual tree growth rates, wood functional traits (e.g., conduit size and density, cell wall thickness and tissue percentage [117]), and isotopic compositions, that can extend back many decades or even centuries. These measurements can be used to test whether or not trees are acclimating to changes in their surrounding environments.

In one study using tree ring measurements to assess patterns of long-term growth in tropical trees, van der Sleen et al. (2015) examined changes in growth rates and water use efficiency in 12 tropical tree species sampled from three lowland rainforest sites (Bolivia, Thailand, and Cameroon). They found significant increases in intrinsic water use efficiency (the rate of moisture loss per unit of carbon gain) in almost all of the study species over the past 150 years as predicted by the “carbon fertilization” hypothesis. They did not, however, find consistent significant changes in site-level tree growth rates [118]. These results are mirrored by findings of dendrochronology studies of other tropical species at other sites [119,120,121], which show that water use efficiency is increasing in many of the tropical sites and species examined to date (with faster increases at drier vs. wetter sites; ref. [122]), whereas tree growth rates show nonsignificant or inconsistent changes [121,123]. One interpretation of these results is that tropical trees are acclimating their water use efficiency (potentially through changes in stomatal traits and stomatal conductances in response to higher CO_2_), which reduces the effects of climate change on growth rates.

Another study by Zuidema et al. (2022) collated tree growth measurements from a new tropical tree-ring network and found that annual tree growth rates increased primarily with dry-season precipitation and decreased with dry-season maximum temperature, and that dry-season climate responses were amplified in regions that are drier, hotter, and more climatically variable [124]. Likewise, a separate tree-ring study showed that the longevity of trees in the lowland tropics decreases markedly at high temperatures, including temperatures likely to soon occur in most lowland tropical forests [88]. Shorter-term studies based on repeated diameter measurements of trees in permanent census plots also show intraspecific declines in tropical tree growth and productivity associated with higher temperatures and changes in vapor pressure deficit or water availability [125,126,127,128]. These patterns all suggest that many tropical trees may not be able to acclimate their growth rates to new climates and that global warming could lead to widespread declines in productivity and increases in tree mortality. More work is needed to assess long-term patterns of growth, water use efficiency, and mortality in more tropical sites and for more tropical species.

Another way that we can look back in time and test for long-term acclimation in individual tropical trees is through the use of herbaria and other historical biological collections [56,129]. To perform a longitudinal study using herbarium specimens, we would need to measure traits on preserved herbarium specimens and then compare these measurements to measurements of the same traits on new samples collected from the same individual source trees [130]. Unfortunately, this is rarely possible because botanical collectors do not typically map or mark their individual source trees. However, during the installation of some permanent tropical forest census plots, collections were made of the mapped and tagged trees to aid with species identifications, and many of the resulting herbarium specimens include the unique tag number of their original source trees. Therefore, by cross-referencing these tag numbers with the most recent plot census data, it should be possible to identify the source trees that are still alive, relocate these source trees within the plots, and then measure a select suite of functional traits on both the historical and modern samples from these trees to test for changes in the traits of individuals through time in relation to local climate change patterns. We are not aware of any such study from the tropics, but this approach was successfully used by Miller Rushing et al. (2009) to test for acclimation of stomatal traits in individual temperate trees growing in the Arnold Arboretum (Boston, MA, USA). As predicted under carbon fertilization, stomatal densities decreased through time to balance CO_2_ and water exchange. However, there were also concurrent increases in stomatal guard cell lengths and thus no net changes in water use efficiency [130]. An important caveat is that the trees included in this study were growing in a managed arboretum and thus they may have been buffered from many abiotic and biotic stressors. Although not at the individual level, a study from the African tropics used historical herbarium samples to show decreases in species’ average stomatal densities but decreasing water use efficiency over the last 80 years [131]. Other analyses of herbarium samples (again not at the individual level) have shown leaf size, and specifically leaf width, to decrease through time in some tree species in response to global warming [132,133].

Although promising, there are clearly many limitations to both the use of tree rings [124,134] and herbarium samples [129]. Both methods are severely limited by a lack of appropriate species (and by over- or under-representation of certain taxonomic and functional groups [135,136,137]), the small number of samples available per species, and the small number of traits that can be measured on each sample since most leaf traits require fresh leaf material and thus cannot be measured on preserved specimens. In addition, these methods can be biased by factors that are hard to control for, such as the effects of ontogeny (age and size) and changing local abiotic and biotic site conditions on leaf and wood traits. Both methods can also suffer from survivorship bias and the over-representation of the individuals and species that can acclimate and tolerate new conditions [137,138,139,140,141,142,143,144]. Despite these and other limitations, tree rings and herbarium samples may be our best tools for assessing the long-term acclimation responses of individual trees to past anthropogenic climate change. In the future, we should also be able to conduct repeat trait censuses of the forests and trees that we are studying now [145,146] to test for any acclimation that is currently underway (towards this aim, we encourage researchers to record sufficient metadata to allow for individual-level resurveys and to make their data and metadata publicly available [147,148]).

Can tropical trees acclimate to rising temperatures? Despite the emergence of new methods, our ability to test for acclimation in individual trees remains limited, and thus we do not yet know if tropical trees can or cannot acclimate to climate change.

Until we can determine if tropical trees can or cannot acclimate, our ability to predict the fate of tropical forests is greatly limited [149]. For example, Feeley et al. (2012) modeled the potential changes of local tree diversity in Amazonian ecoregions under various scenarios of climate change and deforestation and with different assumptions about (1) the ability of tree species to migrate, (2) the effects of rising CO_2_ on water use efficiency, and (3) the ability of species to tolerate higher temperatures through acclimation [150]. By far, the greatest source of uncertainty in the predicted effects of climate change on diversity was the ability of tree species to acclimate or not. If Amazonian trees acclimate to tolerate higher temperatures, then median rates of local tree diversity loss were predicted to be <30% even under the most severe warming and deforestation scenarios (4 °C mean global warming and 22.5% deforestation). This rate was not dramatically impacted by the ability of species to migrate or by the responses of their water use efficiency to CO_2_. If, on the other hand, tropical tree species cannot adequately acclimate to rising temperatures, the median species loss was predicted to exceed 75% even under the most sanguine scenarios of climate change and deforestation (2 °C mean global warming and 13.5% deforestation; under the most severe warming and deforestation scenarios, median species losses without acclimation were 100%). As before, changing the presumed ability of species to migrate and their response to CO_2_ had essentially no effects on the results [150].

Beyond improving our ability to predict the effects of climate change, understanding how tropical trees acclimate or not will also help to improve our predictions of climate change itself. Many of the Earth system models that are used to predict changes in temperature and precipitation incorporate information about vegetation and plant traits [151,152,153,154,155]. To improve the accuracy of these models, it is important to know how traits vary spatially and temporally due to acclimation. Likewise, it is increasingly recognized that better information about thermal acclimation in tropical trees is needed to properly parameterize dynamic global vegetation models [156,157,158].

## 2. Conclusions

Extensive, diverse, and healthy tropical forests are critical to the future of humanity and modern civilization. Unfortunately, it is not yet clear how the many thousands of tree species that comprise tropical forests are responding to climate change, especially in the face of other anthropogenic disturbances such as land use change, habitat loss, and defaunation [159]. Many tropical tree species are ‘migrating’; although migrations were sufficient to offset past climate change, they appear insufficient to keep up with current changes in temperatures and precipitation (especially in the context of other anthropogenic disturbances). Likewise, given the rapid pace and extreme severity of modern climate change, it is unlikely that tropical tree species can adapt (with the caveat that a small number of species may be able to ‘adapt’ by taking advantage of their mutualistic fungal symbionts’ quicker generation times and adaptability, or by switching mutualistic partners). We argue that acclimation is the last and best hope for most tropical tree species to avoid declines in performance and population densities. New methods are being developed to test for individual-level acclimation, but at this point, we still do not know if tropical trees can acclimate or what factors may facilitate or prohibit acclimation. As such, the future of tropical trees—and thus tropical forests—remains uncertain.

## Figures and Tables

**Table 1:** Definitions of key terms used in the review. Note that these definitions may differ in other contexts.

*Acclimation*	Changes in an individual’s behavior, morphology and/or physiology to maintain constant fitness despite changing conditions. Unlike adaptation, acclimation is an individual level response and does not involve changes in an individual’s or population’s genetic makeup.
*Adaptation or Evolutionary Adaptation*	Changes in the relative frequency of genetic alleles in a population or species that increase or maintain fitness due to natural selection.
*Carbon Fertilization*	The hypothesis that increasing concentrations of atmospheric CO_2_ will allow plants to meet their carbon demands faster and with less water loss, leading to increased or accelerated plant growth.
*Climate Analog*	Areas with climate conditions matching those predicted for a focal location.
*Climate Fidelity*	The maintenance of stable climatic niches through time despite climate change, generally achieved through species migrations.
*Dendrochronology*	The use of tree rings to derive temporal information on tree age and performance.
*Dispersal Limitation*	The inability of species migration rates to match climate velocities due to the slow movement of propagules.
*Ecotone*	An abrupt change in habitat structure, composition, or function; may reflect abrupt changes in the underlying environment and/or alternative ecological states.
*Evolutionary Rescue*	When a population or species maintains positive fitness and avoids population decline through evolutionary adaptation.
*Functional Germline*	A slowly dividing but unsegregated lineage of cells that produces both reproductive and somatic tissues.
*In Situ Warming Experiment*	Manipulations of the soil or air temperature around naturally occurring plants in order to simulate future conditions.
*Phenotypic Plasticity*	The ability of an individual genotype to produce different behavioral, morphological and/or physiological phenotypes under different conditions.
*Priority Effect*	An advantage of established individuals or species over later-arriving individuals or species, even if the incumbents would otherwise be at a competitive disadvantage.
*Segregated Germline*	A physical separation of the cells that produce reproductive tissues from those that produce somatic tissues.
*Species Migration*	A shift in a species’ geographic distribution or range through time.
*Thermophilization*	The increased representation of relatively heat-loving or heat-tolerant (i.e., thermophilic) species in a community through time.

## Data Availability

No new data were created or analyzed in this study. Data sharing is not applicable to this article.
